# Allergen-Specific Immunotherapy with Monomeric Allergoid in a Mouse Model of Atopic Dermatitis

**DOI:** 10.1371/journal.pone.0135070

**Published:** 2015-08-14

**Authors:** Nadezda Shershakova, Elena Bashkatova, Alexander Babakhin, Sergey Andreev, Alexandra Nikonova, Igor Shilovsky, Oleg Kamyshnikov, Andrey Buzuk, Olga Elisyutina, Elena Fedenko, Musa Khaitov

**Affiliations:** 1 Department of Nanobiomedical Technology, National Research Center Institute of Immunology, Federal Medical-Biological Agency of Russia, Moscow, Russia; 2 Department of Skin Allergology and Immunopathology, National Research Center-Institute of Immunology Federal Medical-Biological Agency of Russia, Moscow, Russia; 3 Faculty of Natural Sciences, Imperial College London, London, United Kingdom; Université Paris Descartes, FRANCE

## Abstract

Atopic dermatitis (AD) is a widespread and difficult to treat allergic skin disease and is a tough challenge for healthcare. In this study, we investigated whether allergen-specific immunotherapy (ASIT) with a monomeric allergoid obtained by succinylation of ovalbumin (sOVA) is effective in a mouse model of atopic dermatitis. An experimental model of AD was reproduced by epicutaneous sensitization with ovalbumin (OVA). ASIT was performed with subcutaneous (SC) administration of increasing doses of OVA or sOVA. The levels of anti-OVA antibodies, as well as cytokines, were detected by ELISA. Skin samples from patch areas were taken for histologic examination. ASIT with either OVA or sOVA resulted in a reduction of both the anti-OVA IgE level and the IgG1/IgG2a ratio. Moreover, ASIT with sOVA increased the IFN-γ level in supernatants after splenocyte stimulation with OVA. Histologic analysis of skin samples from the sites of allergen application showed that ASIT improved the histologic picture by decreasing allergic inflammation in comparison with untreated mice. These data suggest that ASIT with a succinylated allergen represents promising approach for the treatment of AD.

## Introduction

Atopic dermatitis is a chronic inflammatory skin disease that predominantly affects children and is characterized by skin lesions, persistent erythema, scaling, excoriations, and pruritus. In addition, the disease is commonly associated with allergic rhinitis and asthma. The number of AD patients increased by 10–30% in children and 2–10% in adults in the last 30 years [1–3].

AD is a genetically determined skin disorder of allergic nature with deficiencies in barrier function and specific features of the immune response to allergens characterized by the excessive activation of Th2 lymphocytes and increased production of IgE [4, 5]. These factors lead to an increased prevalence of skin infections in AD patients [6, 7].

Traditional allergen-specific immunotherapy (ASIT) using the re-administration of the allergen in increasing doses causing tolerance to an allergen is known for a long time [8, 9, 10], however, ASIT is not accepted as a therapeutic strategy [11] for AD patients due to concerns about possible skin exacerbation of active AD or potential relapse of latent AD [12, 13]. At the same time the use of ASIT in a randomized, blind, large cohort study of AD-patients (subcutaneous immunotherapy, SCIT) has shown it`s therapeutic efficacy [14]. In an attempt to improve both the efficacy and safety of ASIT, the concept of allergoids (allergens cross-linked with formaldehyde or glutaraldehyde) was introduced in the 1970s and showed good immunogenicity at lower allergenicity in terms of adverse reactions. Recently developed monomeric carbamylated allergens are useful for sublingual immunotherapy (SLIT) as they are resistant to digestion by proteases and rapidly absorbed through the oral mucosa [15, 16]. SCIT with a house dust mite-based allergoid resulted in significant improvements in subjective and objective clinical symptoms of AD patients, combined with serologic and immunologic changes that mirror the therapeutic effect [17]. It is known that allergoids obtained by treatment with aldehydes are randomly cross-linked proteins of high molecular weight [18] and their standardization is very difficult. Furthermore, at local administration, an allergen aggregate can not pass through the mucosa barrier to reach the target cells, unlike the monomeric form of the allergen [15, 19, 20]. The continuous improvement of technologies that results in high-quality extracts and formulations has had a major beneficial impact on both the safety and efficacy of ASIT [21, 22].

Earlier, we have demonstrated that chemical modification of OVA by acylation with N-vinylpyrrolidone-maleic anhydride copolymer or with succinic anhydride leads to a decline in it’s allergenicity, as measured by PCA reaction, RAST inhibition assay and basophil histamine release assay in OVA-sensitive patients [23]. Monomeric succinylated OVA did not induce OVA-specific IgE, however, was capable to activate OVA-specific T-cells to produce IL-2 [24]. In a murine model of bronchial asthma, we have shown that the treatment is effective with both sOVA and non-modified OVA [25]. Succinylated allergens are clearly non-toxic drugs, as it has been recently established that protein succinylation in cells is a normal process of post-translational modification [26]. This reaction is ranked third (after acetylation and ubiquitination) in prevalence among all reactions of post-translational modification of proteins [27]. Currently, thousands of succinylation sites were identified in bacteria, yeast, human cells, and animal tissues, demonstrating widespread succinylation in diverse organisms and modification of proteins may occur either enzymatically or non-enzymatically with succinyl-CoA [28].

The aim of this study was to evaluate the therapeutic efficiency of experimental ASIT with both non-modified OVA and succinylated OVA in a mouse model of AD [29] and to determine the rationale of involving the modified allergen in AD therapy.

## Materials and Methods

### Sensitization of mice and ASIT protocol

Female BALB/c mice aged 4–6 weeks were purchased from the animal nursery Filial SCBMT “Stolbovaya” (Moscow region, Russia) and kept in a pathogen-free environment with an OVA-free diet. All experimental procedures were carried out in strict accordance with the recommendations in the Guide for the Care and Use of Laboratory Animals of the Ministry of Health of the Russian Federation (Permit Number: 708) and “Regulations on the ethical attitudes to laboratory animals of NRC Institute of Immunology FMBA of Russia” (Moscow, Russia). The study protocol was reviewed and approved by the Bioethic Committee of NRC Institute of Immunology FMBA Russia (Permit Number: 01385). Mice were quarantined for ≥7 days before study initiation. Eight mice in each group were utilized to minimize the number of experimental animals needed while ensuring the validity of statistical power. Each mouse group was housed in a separate plastic cage in a temperature-controlled environment and given feed and water *ad libitum* using a 12-h light—dark cycle. They were fed an OVA-free diet and given water ad libitum.

EC sensitization of mice was done as described by Spergel et al. [30]. Briefly, mice were shaved with an electric razor. OVA (100 μg; Sigma, USA) in PBS (100 μl) or placebo was placed on a 1x1 cm^2^ patch of sterile gauze, which was then secured onto the skin with a transparent bioclusive dressing (Systagenix Wound Management Limited, United Kingdom). The patch was applied thrice over a 1-week period. An inspection at the end of each sensitization period confirmed that the patch had remained in place.

ASIT was done by a SC injection with increasing doses of: non-modified OVA; OVA modified by succinylation (85% of modification) or sham treated with PBS, between 1^st^ and 2^nd^ OVA-applications. Animals were anesthetized with diethyl ether and then sacrificed.

### Succinylation of OVA

Succinylation of OVA was done as described previously [31–33] with a slight modification. Crystalline succinic anhydride (100 mg) was added slowly to a 2–5% aqueous solution of OVA (100 mg) with vigorous stirring at room temperature and pH 8.5–9.0. After pH stabilization, the solution obtained was extensively dialyzed against distilled water, filtrated through a 0.2-micron filter, and lyophilized. The degree of succinylation was determined by the TNBS method previously described by Habeeb [34] with a slight modification. The absorbance at 420 nm was recorded (spectrophotometer Cary 100, Agilent) against the solution of TNBS as blank; the absorbance of non-modified OVA was set to 100% free amino groups. For greater accuracy, we used 5 dilutions of the proteins. Solutions (1 ml) of OVA and sOVA in 0.2M borate buffer (pH 10.2) at concentrations of 1, 0.5, 0.25, 0.125, and 0.0625 mg/ml were each mixed with 1 ml TNBS solution (0.5 mg/ml of distilled water) and then incubated for 20–30 min. An error in determining the extent of modification may vary about 2%, since there were variations from batch to batch under identical conditions. The absorbance was measured at 420 nm against a blank prepared by mixing 1 ml of borate buffer with 1 ml TNBS.

### Specific IgE, IgG, IgG1, and IgG2a Levels in Serum

Sera were collected before, during, and after ASIT. In this study we did not use commercial kits to measure quantitatively anti-OVA mouse IgE. Rat anti-mouse IgE (BD, Cat.553413) was used for coating the plates and mouse anti—ovalbumin mAb (AbD Serotec, UK) and biotin OVA was used for detection. These components were used to construct the calibration curve and then to analyses sera. The levels of anti-OVA IgG, IgG1 and IgG2a antibodies in sera were detected by ELISA (ELISA kits from BD, USA) according to the manufacturer’s protocol.

### Cytokine assay

The spleens were taken after the last allergen application aseptically. Splenocytes (5×10^6^ cells/well) were plated in 12-well plates and incubated for 72 h in RPMI 1640 medium (GIBCO, USA) containing 10% FBS (HyClone, USA), gentamicin (800 μg/mL), 50 μM β-ME at 37°C in a 5% CO_2_ humidified incubator. Splenocytes were stimulated or non-stimulated with OVA (4 μg/mL). The levels of IL-4, IL-5, IL-17, IFN-γ, and IL-12 (p40) in supernatants of OVA-stimulated or non-stimulated splenocytes were determined by ELISA (Duo-Set, R&D Systems, UK; ELISA set, BD, USA) according to the manufacturer’s protocol.

### Real-time PCR analysis (qRT-PCR)

We collected the OVA-stimulated or non-stimulated splenocytes from each mouse and stored them at -80°C until use. The total RNA from OVA-stimulated mice splenocytes was extracted using the RNeasy Mini Kit (Qiagen, Courtaboeuf, France) according to the manufacturer’s instructions. The RNA concentration was determined, and cDNA was synthesized through a reverse transcription reaction (Reverta-L, Interlabservice, Moscow, Russia). The reverse transcription reaction product was amplified in a qRT-PCR using an iCycler iQ Real-time PCR Detection System (Bio-Rad Laboratories, USA) and the PCR-Mix kit (Sintol, Russia). The following primers and probe were used for mouse Foxp3 mRNA quantification by qRT-PCR: forward 5’- TCACCTATGCCACCCTTATC, reverse 5’–TTCTGAAGTAGGCGAACATG, probe 5’- CGGAGAGGCAGAGGACACTCAATG.

We normalized real-time PCR assays to mouse HPRT: forward 5’- TATACCTAATCATTATGCCGAGGAT, reverse 5’–CTTTAATGTAATCCAGCAGGTCAG, probe 5’- TCCTCATGGACTGATTATGGACAGGACT.

The results are presented as mRNA expression. Calculations to determine the relative level of gene expression were made using the ΔCq method and referring to the mHPRT in each sample; the results are presented as arbitrary units.

### Histopathological Analysis

The skin specimens from patch areas were removed for histologic examination immediately after the last EC application of OVA. Skin biopsies were taken from similar body sites, fixed overnight with 10% paraformaldehyde at 4°C, and embedded in paraffin. Four-micrometer sections were stained with hematoxylin and eosin (H&E). The histologic preparations were analyzed under a light microscope (Leica DM2000, Germany) with a 50x, 100x, and 400x magnification.

Epidermal thickening, epidermal necrosis, epidermal hyperkeratosis, dermal and subcutaneous fat necrosis, swelling, hemorrhage, and cell infiltration of dermis and subcutaneous fat were the main assessment criteria for histologic skin lesions (Table 1).

**Table 1:** The main assessment criteria for histologic skin lesions.

Criterion	The degree of manifestation	Score
epidermal thickening	absent	0
	mild	1
	moderate	2
	pronounced	3
epidermal necrosis	absent	0
	present	1
epidermal hyperkeratosis	absent	0
	present	1
connective tissue like dermal proliferation	absent	0
	mild	1
	moderate	2
	pronounced	3
dermal necrosis	absent	0
	present	1
dermal swelling	absent	0
	present	1
dermal hemorrhage	absent	0
	present	1
connective tissue like subcutaneous fat proliferation	absent	0
	mild	1
	moderate	2
	pronounced	3
subcutaneous fat necrosis	absent	0
	present	1
subcutaneous fat swelling	absent	0
	present	1
subcutaneous fat hemorrhage	absent	0
	present	1
cell infiltration of dermis	absent	0
	mild	1
	moderate	2
	pronounced	3
cell infiltration of dermis (eosinophils)	absent	0
	mild	1
	moderate	2
	pronounced	3
cell infiltration of dermis (polynuclear leukocytes)	absent	0
	mild	1
	moderate	2
	pronounced	3
cell infiltration of dermis (lymphocytes)	absent	0
	mild	1
	moderate	2
	pronounced	3
cell infiltration of subcutaneous fat	absent	0
	mild	1
	moderate	2
	pronounced	3
cell infiltration of subcutaneous fat (eosinophils)	absent	0
	mild	1
	moderate	2
	pronounced	3
cell infiltration of subcutaneous fat (polynuclear leukocytes)	absent	0
	mild	1
	moderate	2
	pronounced	3
cell infiltration of subcutaneous fat (lymphocytes)	absent	0
	mild	1
	moderate	2
	pronounced	3

Each parameter had the degree of manifestation and appropriate score. The histological inflammatory index was calculated by averaging the sum of these scores.

### Statistical Analysis

The data are shown as means ± SE. Statistical analysis was carried out with the program Statistica 8.0 (StatSoft Inc., USA). The significance of the results was determined by a Student’s t test. Differences were considered significant at p<0.05.

## Results and Discussion

### Sensitization of mice to model AD and OVA modification

**Fig 1:**
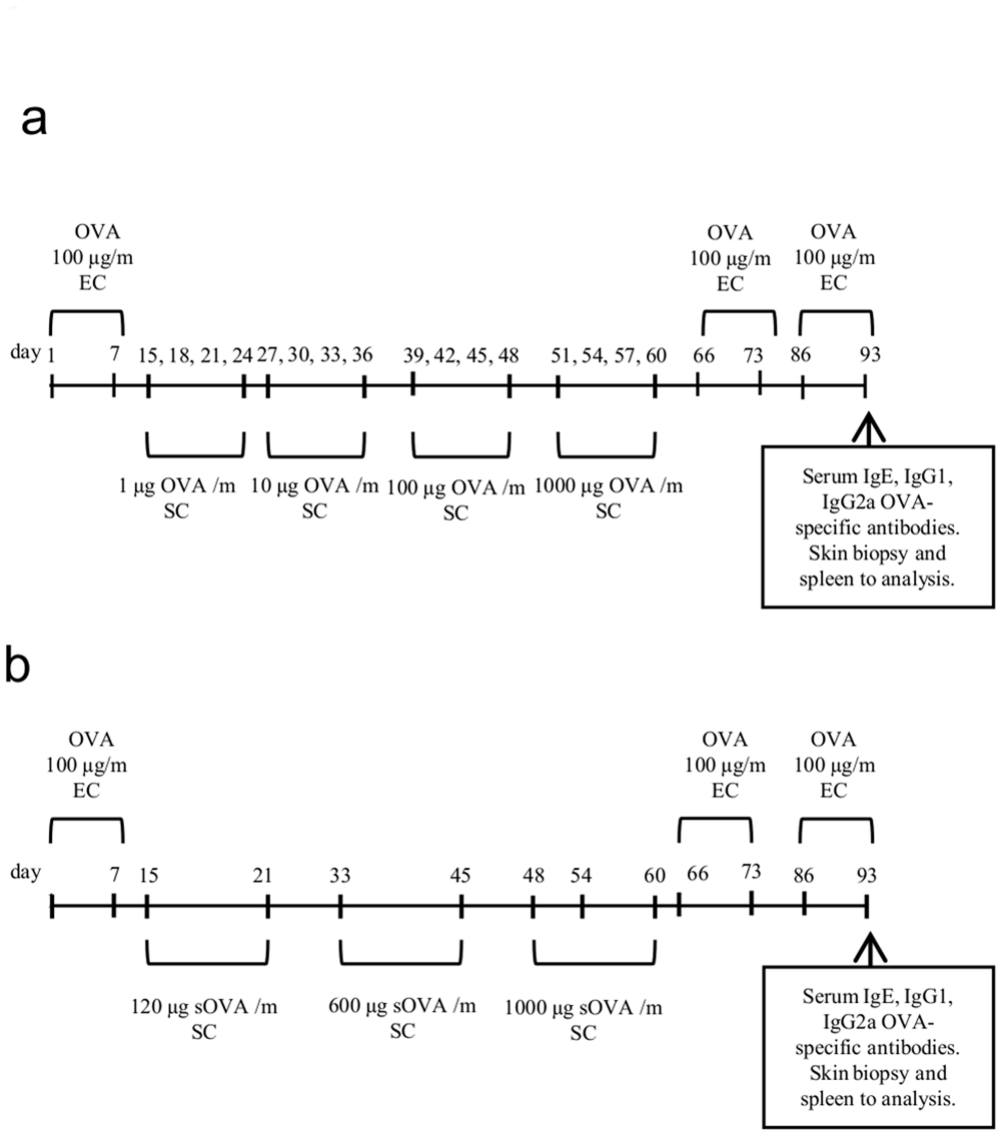
Protocol of sensitization and ASIT with OVA or sOVA. Mice were sensitized with OVA (100 μg) or saline applied at 100 μl to a sterile patch. The patch was applied for 1-wk and then removed. ASIT was performed between the 1^st^ and 2^nd^ OVA applications by SC injection of increasing doses of OVA (a), sOVA (b), or PBS (as control). After ASIT, the mice had two 1-wk exposures to a patch separated by 2-wk intervals. The control group received PBS at the same time.

Female BALB/c mice were epicutaneously sensitized with an OVA solution (1 mg/ml) during three one-week exposures with two-week intervals (Fig 1). Sensitized mice had elevated levels of anti-OVA IgE and IgG1. In addition, it was demonstrated that the levels of IL-4 and IL-5 in supernatants of OVA-stimulated murine splenocytes from sensitized mice were higher than that of control (naïve mice). Histological analysis of specimens taken from the patched skin revealed that epicutaneous (EC) sensitization led to a local allergic inflammation in the skin. The dermal layer of OVA sensitized mice was infiltrated with neutrophils, lymphocytes, mast cells, and eosinophils. Collagenosis and keratosis in the same areas of the skin were also revealed. Thus, we concluded that EC sensitization led to a local allergen-specific inflammation that is similar to that of human AD regarding clinical, histologic and immunologic features.

Succinylated OVA used as the therapeutic allergen was prepared as described in Materials and Methods. The quantitative determination of free amino groups in sOVA by the TNBS-method showed almost total modification (98–100%) of lysine Ɛ-amino groups in the protein.

Experimental ASIT was performed as described in Materials and Methods.

### Effect of experimental ASIT on specific IgE and IgG levels

**Fig 2:**
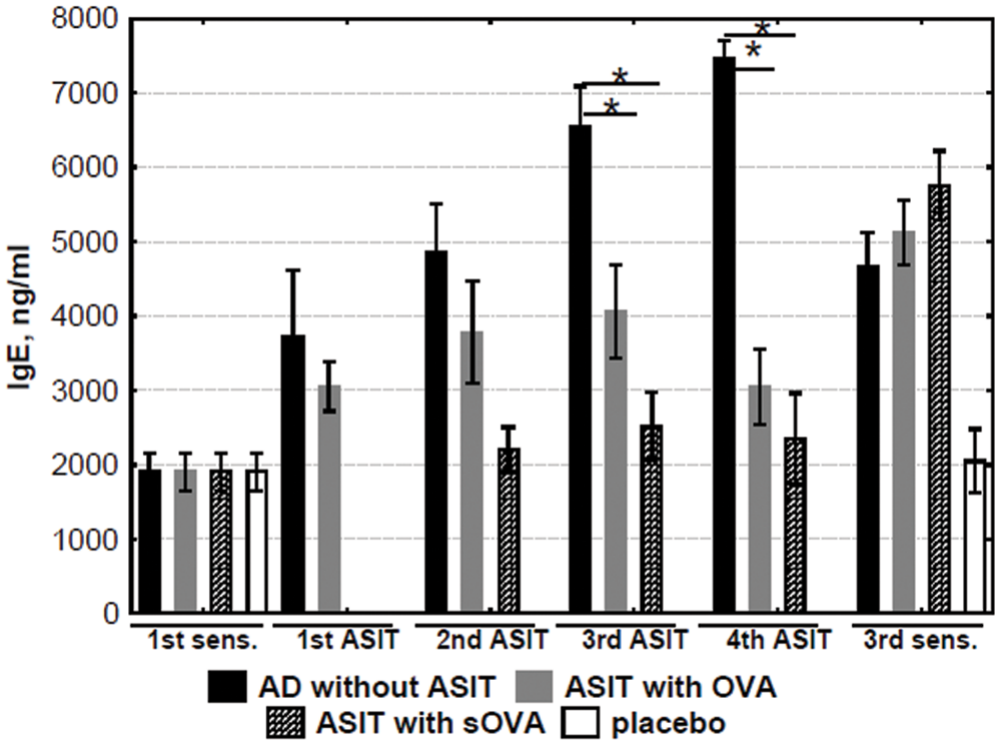
Analysis of OVA-specific IgE. The levels of IgE in sera obtained before, during, and after ASIT were detected and quantified by ELISA. The results are presented as mean IgE concentration (mean±SE, n = 8 for each). AD without ASIT: OVA-sensitized untreated mice; ASIT with OVA: OVA-sensitized mice treated by ASIT with OVA; ASIT with sOVA: OVA-sensitized mice treated by ASIT with sOVA; placebo: PBS-sensitized mice. (*, p<0.05).

OVA-specific antibody levels of the IgE isotype were significantly increased in sensitized mice (group “AD without ASIT”) but not in mice sham-sensitized with PBS (control mice, group “placebo”). The anti-OVA IgE level in “ASIT with OVA” and “ASIT with sOVA” groups gradually decreased during ASIT (Fig 2). However, the levels of specific IgE in the treated and non-treated mice groups did not differ from each other significantly after the last application of OVA (3^rd^ sensitization).

**Fig 3:**
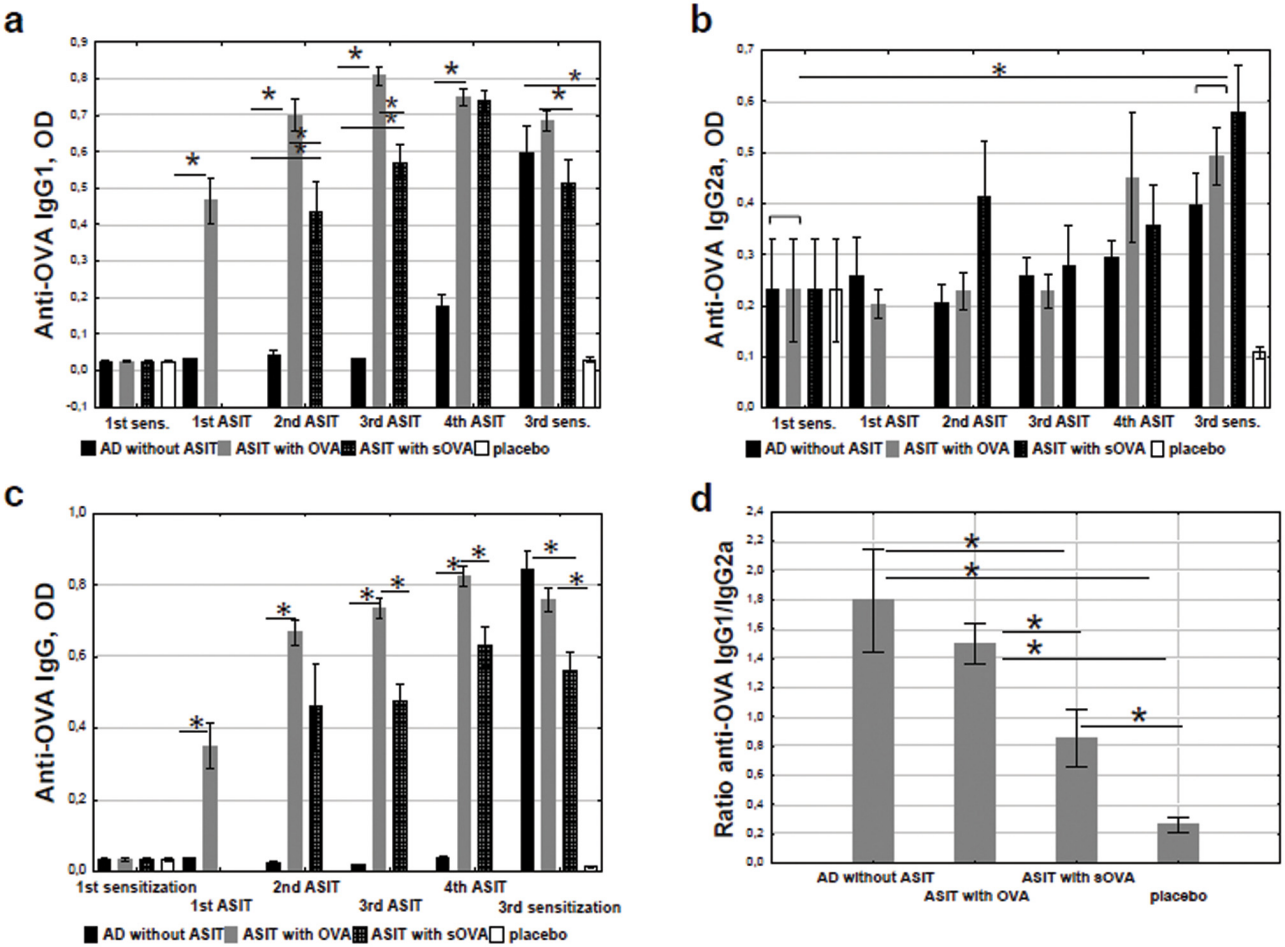
Analysis of OVA-specific IgG. The levels of antibodies in sera obtained before, during, and after ASIT were detected and quantified by ELISA. The results are presented as mean antibodies concentrations (mean±SE, n = 8 for each). AD without ASIT: OVA-sensitized untreated mice; ASIT with OVA: OVA-sensitized mice treated by ASIT with OVA; ASIT with sOVA: OVA-sensitized mice treated by ASIT with sOVA; placebo: PBS-sensitized mice. (a) anti-OVA IgG1 response in mice with ASIT, (b) anti-OVA IgG2a response in mice with ASIT, (c) anti-OVA IgG response in mice with ASIT, (d) ratio of OVA-specific IgG1/IgG2a antibody in mice with ASIT, (*, p<0.05)

The level of specific IgG1 was not significantly increased during ASIT (Fig 3a). It should be noted that the final anti-OVA IgG1 level was significantly decreased in the group “ASIT with sOVA” compared with the group “ASIT with OVA.” In addition, the final OVA-specific IgG2a response was significantly increased after ASIT in comparison with it`s level before the therapy (Fig 3b). Herewith, the IgG2a level in the group “ASIT with sOVA” compared with the group “ASIT with OVA” did not differ. There was no significant difference between IgG2a levels after the 1-st or final sensitization in group “AD without ASIT”.

It was shown that the anti-OVA IgG level was increased during ASIT in group “ASIT with OVA”, but not “ASIT with sOVA” (Fig 3c). In addition, the final OVA-specific IgG response in “ASIT with sOVA” was significantly decreased after ASIT in comparison with “ASIT with OVA” group. Thus, final anti-OVA IgG and IgG1 levels were significantly decreased, and the specific IgG2a level was increased in “ASIT with sOVA” in comparison with OVA-treated group.

Mean IgG1 to IgG2a antibody ratios were used as an indicator of Th1 or Th2 bias in immune responses [35]. The ratio of specific IgG1/IgG2a was decreased in both the OVA- and sOVA-treated groups (Fig 3d). More importantly a reduction in the ratio was observed in the group “ASIT with sOVA,” indicating a shift toward Th1 immune response.

### Suppression of allergen-related cytokines by ASIT

**Fig 4:**
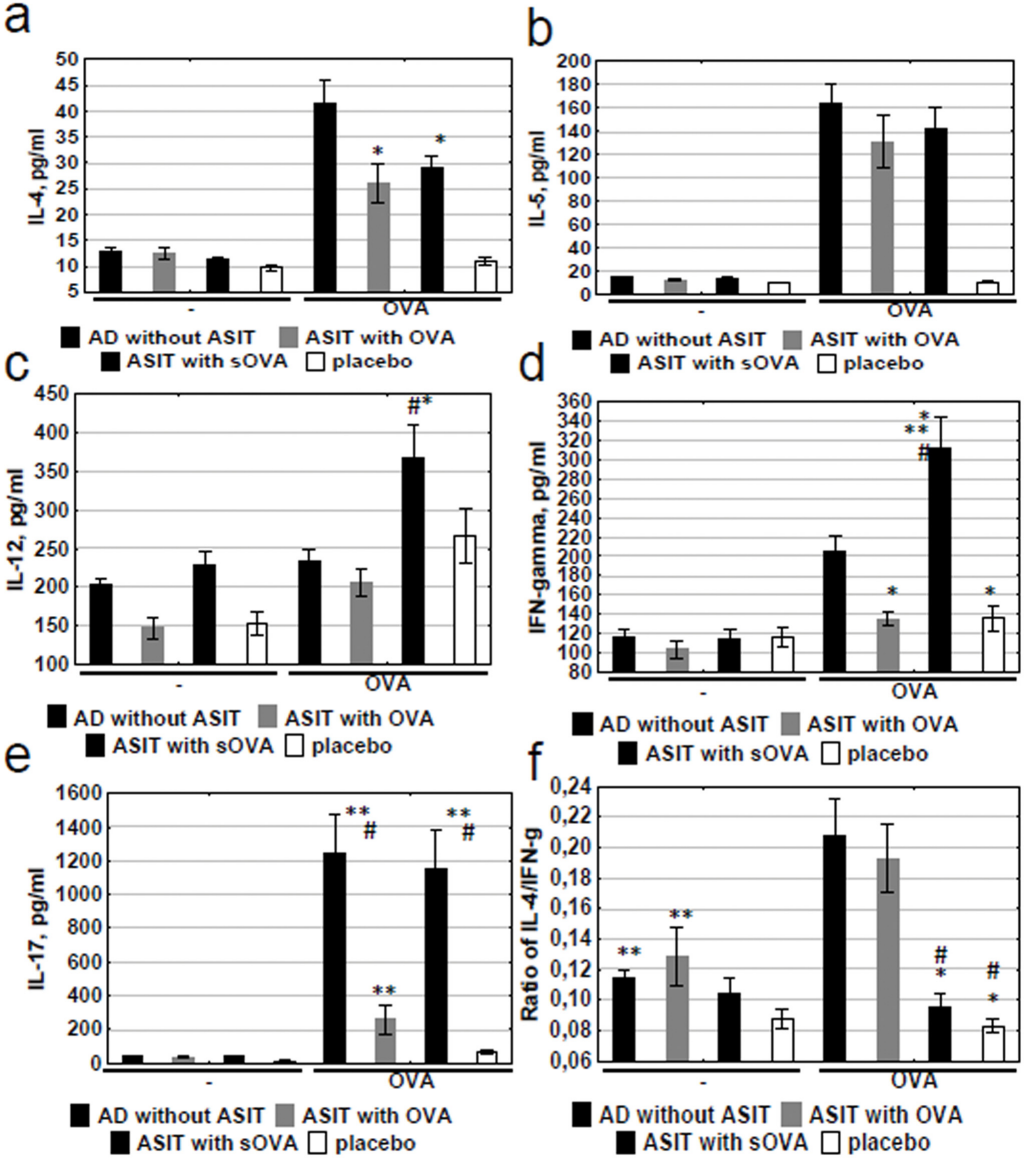
Levels of cytokines. The levels of cytokines in supernatants of OVA-stimulated mice splenocytes (incubated with OVA for 72hrs) were quantified by ELISA. The results are presented as mean IL concentration (mean±SE, n = 8 for each). AD without ASIT: OVA-sensitized untreated mice; ASIT with OVA: OVA-sensitized mice treated by ASIT with OVA; ASIT with sOVA: OVA-sensitized mice treated by ASIT with sOVA; placebo: PBS-sensitized mice. Level of (a) the IL-4, (b) IL-5, (c) IL-12, (d) IFN-γ, and (e) IL-17; (f) IL-4/IFN-γ ratio in mice with ASIT. *, p<0.05 versus “AD without ASIT”; **, p<0.05 versus “placebo”; #, p<0.05 versus “ASIT with OVA”.

The expression of cytokines relevant for a Th1, Th2, or Th17 immune response was evaluated after the last application of OVA in supernatants of OVA-stimulated mice splenocytes. IL-4 is crucial for the initiation of allergic disease because it stimulates the differentiation of uncommitted Th cells toward a Th2 phenotype and inhibits Th1 differentiation. The expression of IL-4 significantly dropped during ASIT with both modified and non-modified allergens (Fig 4a). There was no difference in IL-4 levels between the groups “ASIT with sOVA” and “ASIT with OVA.”

IL-5, as well as IL-4, is associated with allergic inflammatory pathology, including bronchial asthma and AD. In particular, this cytokine provides differentiation, recruitment and survival of eosinophils, elevated IL-5 expression in broncho-alveolar lavage was detected in asthma patients. In our case, ASIT performed with OVA and sOVA slightly reduced IL-5 concentration (Fig 4b), however it`s level in the treated groups did not significantly differ from that in the non-treated group, i.e. ASIT effect on the production of IL-5 was significantly weaker as compared to IL-4.

We also assessed the levels of IL-12 and IFN-γ which are important markers of a Th1 immune response. The highest concentration of IL-12 was detected in the group “ASIT with sOVA” (Fig 4c). Furthermore, the concentration of IFN-γ during ASIT was shown to be significantly higher in the same group (Fig 4d).

Epicutaneous immunization of mice with OVA has been shown to trigger activation of IL-17 producing T cells in the draining lymph nodes and spleen [36], and IL-17 has been found to be associated with acute AD skin lesions [37]. In addition, the development of bacterial infection has been reported to be associated with the Th17 immune response [38]. In our experiments, we found that the level of IL-17 was increased in the AD group. However, the IL-17 concentration was significantly decreased during ASIT with the non-modified allergen compared with both the “AD without ASIT” and the “ASIT with sOVA” group (Fig 4e). These data suggest that ASIT with OVA leads to an alteration in the Th17 immune response, which in turn can affect the level of Th2 cytokines.

Both Th1 and Th2 cytokines (as INF-γ and IL-4) are known to take part in Th17 cell development and proliferation, and their suppression causes an increase in the number of Th17 cells [39]. Rui He *et al*. [40] showed that epicutaneous sensitization in the absence of IL-4/IL-13 induces an exaggerated Th17 response. We observed that the level of IL-4 in the group “ASIT with sOVA” was significantly decreased but IFN-γ was significantly increased in comparison with “AD without ASIT.” These data partially explain why the level of IL-17 did not change in the sOVA-treated mice. In contrast, IL-4 and IFN-γ expression was suppressed in the group “ASIT with OVA.” Therefore, ASIT with sOVA does not affect the Th17 immune response, but OVA treatment suppresses the development of the latter. It should be noted that the purulent inflammation in mice treated with sOVA was noticeably less severe compared with that in OVA-treated mice or the “AD without ASIT” group. These data explains the decrease and the increase in IL-17 levels in the groups “ASIT with OVA” and “ASIT with sOVA,” respectively.

The IL-4/IFN-γ ratio was at it`s maximum and approximately equal in the “ASIT with OVA” and model groups but was at it`s minimum in the groups “ASIT with sOVA” and “placebo” (Fig 4f). These values confirm the assumption that ASIT with OVA can downregulate the Th17 immune response. In contrast, ASIT with sOVA did not influence the Th17 production; however, we observed a polarization of the immune response toward the Th1 profile. Thus, sOVA treatment suppressed the Th2 immune response and reduced the allergic inflammation in AD mice.

### Induction of Foxp3

Both clinical trials and experimental models have shown that specific immunotherapy increases the production of local and systemic Foxp3+ T_regs_ [41, 42]. Therefore, the increase in T_regs_ caused by ASIT may establish a global tolerance; previous studies have shown an association between ASIT and the induction of T_regs_ [43].

**Fig 5:**
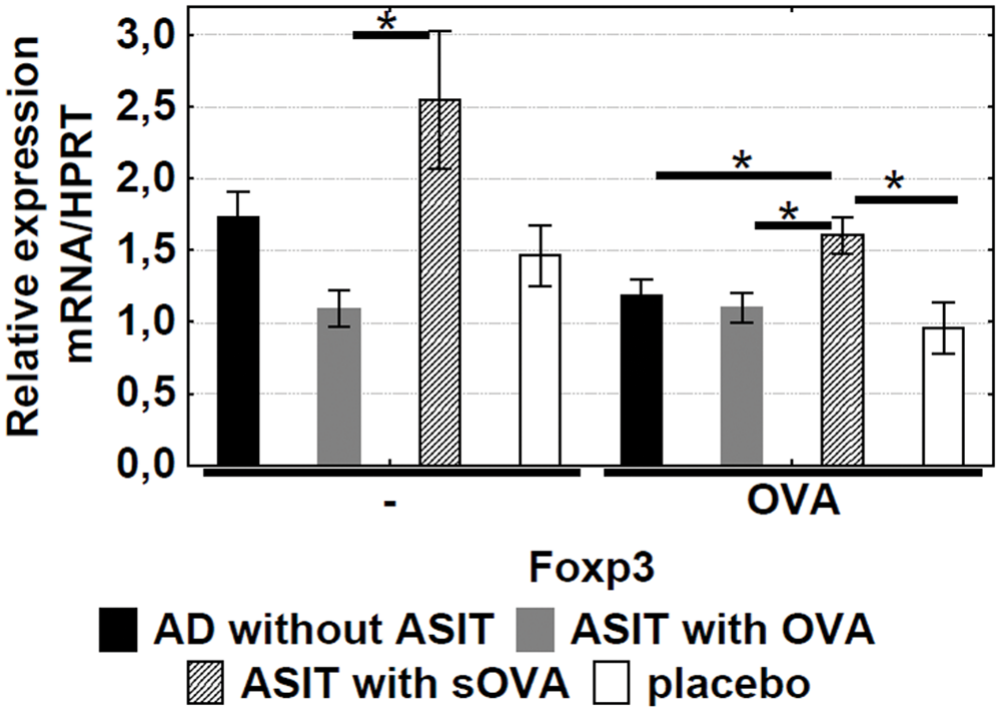
Level of Foxp3 expression. The specific mRNAs in OVA-stimulated mice splenocytes (incubated with OVA for 72hrs) were quantified by qRT-PCR. The results are presented as mean mRNA expression (mean±SE, n = 8 for each). The relative levels of Foxp3 expression were calculated by referring to the HPRT (hypoxanthine guanine phosphoribosyltransferase) in each sample. AD without ASIT: OVA-sensitized untreated mice; ASIT with OVA: OVA-sensitized mice treated by ASIT with OVA; ASIT with sOVA: OVA-sensitized mice treated by ASIT with sOVA; placebo: PBS-sensitized mice. *, p<0.05.

The expression of Foxp3 mRNA in OVA-stimulated mice splenocytes was higher in the group “ASIT with sOVA” than in the other groups (p<0.05) (Fig 5), suggesting a role of T_regs_.

The enhanced activity of allergen-specific Foxp3+ T_regs_ after ASIT might point to the induction of a tolerance mechanism. These findings indicate that ASIT with sOVA is a more effective therapy than ASIT with non-modified OVA.

### Histological assay

**Fig 6:**
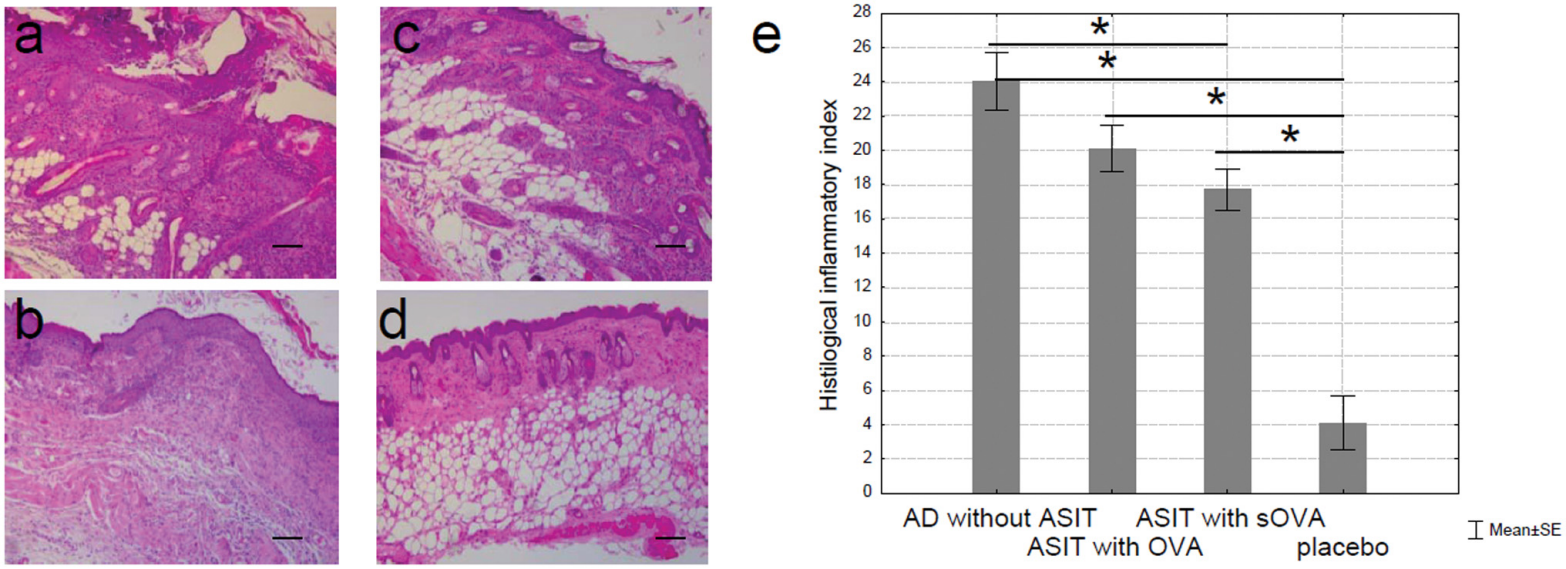
Histologic features of OVA- and sOVA-treated skin sites in BALB/c mice. (a) Mice sensitized with OVA and treated with PBS (“AD without ASIT”), (b) mice sensitized with OVA and treated with OVA (“ASIT with OVA”), (c) mice sensitized with OVA and treated with sOVA (“ASIT with sOVA”), and (d) mice sensitized with PBS (“placebo”). Skin sections were stained with H&E and examined at 100x. Scale bars 100 μm. There is marked hyperplasia of the epidermis, a dermal infiltrate (a-c). The cellular infiltrate consists of neutrophils, eosinophils, and lymphocytes. (e) Summary index of the main assessment criteria for histologic skin lesions.

Epicutaneous OVA sensitization leads to local inflammation of the skin. To determine the degree of local inflammation in treated and non-treated mice, histologic analysis of patched skin was performed. Skin biopsies from sensitized mice were taken after the final series of sensitization. We observed a pronounced epidermis thickening with acanthosis (diffuse epidermal hyperplasia) at the site of EC sensitization with OVA (“AD without ASIT” group) but not with saline (“placebo” group) (Fig 6a and 6d). Necrotic masses of keratin and white blood cells were found on the acanthosis surface. There were pronounced diffuse mixed leukocyte infiltrates with a moderate number of eosinophils in dermal and subcutaneous fat. Skin appendages, such as hair follicles and sebaceous glands, were retained in the lesion area and had degenerative changes.

Fig 6b shows that the epidermis was moderately thickened (“ASIT with OVA” group) due to a spinous layer, on which hyperkeratosis was observed. In addition, there were moderate diffuse mixed leukocyte infiltrates with a small amount of eosinophils in dermal and subcutaneous fat. Skin appendages were partially retained. It should be noted that only in group “ASIT with OVA” there was a clear visible fibrous process with replacement of adipose to connective tissue in subcutaneous fat. Also, as in the case of AD, the skin of animals sensitized to OVA, but not to saline, was infiltrated with neutrophils, lymphocytes, mast cells, and eosinophils. In some areas, the infiltrates penetrated into subcutaneous fat. In addition, purulent inflammation was observed in the AD group and partially in the group “ASIT with OVA.” Epidermal thickening, epidermal necrosis, epidermal hyperkeratosis, dermal and subcutaneous fat necrosis, swelling, hemorrhage, and cell infiltration of dermis and subcutaneous fat were the main assessment criteria for histologic skin lesions. Skin inflammation was evaluated in a semi-quantitative scale. The histologic inflammatory index was calculated by averaging the sum of scores. In contrast, in the group “ASIT with sOVA” only slight thickening of the epidermis, and stratification of keratinocytes was observed and retained until the end of the study. In addition, mild leukocyte infiltrates with lymphocytes only in dermal and subcutaneous fat were observed with skin appendages retaining intact (Fig 6c and 6e). No pathologic changes were observed in the “placebo” group.

The maximum inflammatory index was observed in the AD group (24 points) while the minimum was observed in the “placebo” group (4.1 points). The histologic inflammatory indexes for the groups “ASIT with OVA” and “ASIT with sOVA” were 20.1 and 17.7 respectively.

Assessment of pathological skin condition in experimental mouse groups demonstrated that the most severe lesions were found in the group “AD” that generally corresponds to a typical allergic inflammation characteristic of AD. The histologic picture was found to be improved significantly in the group “ASIT with OVA” and histopathological changes were almost completely absent in the group “ASIT with sOVA.” In the “placebo” group no pathologic changes were presented. Thus, the histologic analysis of skin samples from the sites of allergen application revealed that ASIT, with both OVA and sOVA, improved the histologic picture by reducing the allergic inflammation by approximately 16 and 26%, respectively, compared with the untreated mice.

The allergic reactivity of proteins is known to be determined by B-cell epitopes capable of binding to IgE molecules. As a rule, IgE-binding epitopes are conformation-dependent [44]. Therefore, damage in protein conformation usually leads to a reduction in allergenicity. Hence, the principal approach to ASIT is to modify B-cell epitopes to prevent IgE binding. However, T-cell epitopes should be preserved to some extent to retain the capacity to induce the T-cell response.

Previously we have shown that modification of OVA by succinylation led to inhibition of allergen histamine release activity as it was tested on basophils of OVA-sensitive patients. IgE-binding activity of sOVA was also significantly reduced as it was shown by RAST inhibition test. sOVA stimulated OVA-specific T cells to produce cytokine release at the same level as in the case of non-modified OVA. Thus succinylation of allergens markedly reduces their allergenicity while preserving immunogenicity [45]. We also assume that the modification of lysine residues of the allergen can partially change the character of it`s processing in antigen-presenting cells (APCs) and thereby modify the peptide/MHC repertoire for presentation to T cells. A lysine succinylation in polypeptides has been shown to reduce the rate of hydrolysis of adjacent peptide bonds to 1–2 orders of magnitude, depending on the amino acid environment [46, 47].

In the current study we demonstrated that ASIT is an effective approach in the treatment of AD and suppression of the Th2 responses based on the decrease in skin lesions, as well as improvement of immunologic and histologic features. However as a drug for ASIT, sOVA was shown to be more effective than OVA. The concordant decline in anti-OVA IgG1, IL-4, and IL-5 and the increase in IL-12 and IFN-γ after ASIT with the modified allergen indicated a clear switch from Th2 to Th1 type of immune response. Importantly sOVA treatment retained the affected skin histologic picture almost intact. Aside from being an effective treatment for experimental AD, data obtained in this study indicate that ASIT with succinylated allergens is a perspective approach for the treatment of AD.
